# Near-Infrared Spectroscopy Non-Destructive Detection Modeling for Starch Content in Kernels of 58 Rainfed Corn Varieties

**DOI:** 10.3390/foods15152599

**Published:** 2026-07-24

**Authors:** Xiaoguang Yan, Guoliang Wang, Zhiyuan Ma, Liting Qi, Yanwei Du

**Affiliations:** Institute of Millet Research, Shanxi Agricultural University, Changzhi 046000, China; gzsyxg001@sxau.edu.cn (X.Y.); wangguoliangwz@sxau.edu.cn (G.W.); mazhiyuan1713@sxau.edu.cn (Z.M.); qiliting@sxau.edu.cn (L.Q.)

**Keywords:** corn kernels, near-infrared hyperspectroscopy, starch content, key wavelengths, predictive modeling

## Abstract

Traditional methods for determining starch content in corn kernels are labor-intensive, destructive, and inefficient. To overcome these challenges, this work developed a rapid, non-destructive approach based on near-infrared hyperspectral imaging, applied to 58 rainfed corn varieties. A spectral preprocessing scheme combining wavelet transform, multiplicative scatter correction, and standard normal variate transformation was employed to enhance spectral quality. A two-stage wavelength selection framework was established using competitive adaptive reweighted sampling and sparrow search algorithm optimization. From the selected optimal wavelengths, four predictive models, namely partial least squares regression, artificial neural network (ANN), convolutional neural networks, and gradient boosting decision tree, were established, implemented, and systematically compared. The results identify 14 key wavelengths (1020.65–1647.71 nm) strongly correlated with starch content, with clear assignments to specific chemical bonds and good physical interpretability. Among these models, the ANN exhibited the best performance. The R^2^, RMSE, and RPD of the test set were 0.826, 0.759%, and 2.40, respectively, indicating favorable prediction accuracy and generalization ability. These key wavelengths provide a foundation for developing portable detection instruments. This work supports corn quality grading, breeding of high-starch varieties, and rapid raw material screening, thereby enhancing the quality and efficiency of the corn industry.

## 1. Introduction

As one of the world’s most vital food crops, corn is not only a primary energy source for the human diet but also a critical raw material for industrial starches, livestock feed, and bioenergy production [1,2,3]. Starch is the most abundant component in corn kernels, and its content directly determines processing quality, economic value, and industrial end-use applications [4,5,6]. High-starch corn varieties provide higher starch extraction yields and better economic returns in wet-milling processes, while low-starch varieties may have advantages in other quality attributes, such as protein and oil content [5]. Therefore, accurately determining starch content differences among corn varieties and establishing rapid, efficient quality detection methods are of significant theoretical value and practical importance for germplasm screening, variety breeding, and the graded utilization of corn.

Traditional methods for determining starch content have relied primarily on chemical analysis techniques, such as DNS colorimetry [7,8,9] and acid hydrolysis [7,9,10]. Although these methods offer high accuracy and reliability, they are labor-intensive, time-consuming, and require large volumes of chemical reagents. Furthermore, they are destructive to samples, which limits their suitability for rapid, non-destructive, and high-throughput analysis—critical needs in large-scale breeding screenings and industrial in-line detection. Near-infrared (NIR) spectroscopy has been extensively applied in agricultural product quality assessment, owing to its distinct advantages including rapid analysis, minimal sample preparation, non-destructive measurement, and the capacity for simultaneous quantification of multiple chemical components. These attributes make NIR spectroscopy a highly efficient and cost-effective tool for large-scale screening and real-time quality monitoring in the agricultural sector. It reflects the overtone and combination absorption information of hydrogen-containing groups, such as C-H, O-H, and N-H, within the sample [11]. As a polysaccharide, starch is rich in hydroxyl groups and possesses a carbon–hydrogen backbone. These structural features provide a theoretical foundation for using NIR spectroscopy to rapidly determine starch content in corn.

NIR spectral data, however, suffer from high dimensionality, extensive redundancy, and severe collinearity. The raw spectra contain both target-related chemical signals and extraneous interferences. Specifically, the physical state of the sample, including particle size, bulk density, and surface scattering, can significantly distort spectral signals by altering the light propagation path within the sample. In addition, unavoidable instrument noise in NIR detection systems, arising from electronic fluctuations and environmental factors, further complicates the extraction of effective information. Moreover, other inherent components in corn, such as moisture, protein, and fat, exhibit overlapping absorption bands with starch in the NIR region. This overlap leads to mutual interference, reducing the spectral specificity for starch content determination. Due to the complexity and diversity of these interfering factors, a single spectral preprocessing method is often insufficient to comprehensively eliminate all types of interference [12]. Traditional wavelength selection methods, such as the correlation coefficient method and stepwise regression, often struggle to effectively handle high-dimensional, collinear spectral data [13]. The correlation coefficient method tends to select wavelengths that have low correlation with the target component but high linear correlation among themselves. This leads to information redundancy and reduces the accuracy of corn quality detection models. Stepwise regression is prone to local optimization when processing high-dimensional near-infrared data and fails to fully eliminate collinearity, thereby compromising the stability of subsequent predictive models [14]. The competitive adaptive reweighted sampling (CARS) algorithm [15], which combines Monte Carlo sampling with partial least squares (PLS) coefficients [16], has been widely applied in spectral feature selection due to its ability to rapidly identify important variables from a large set of wavelengths. However, studies have found that the selection results of CARS may still retain redundant information, as the algorithm primarily focuses on the importance of individual variables while neglecting interactions among them. Furthermore, due to the randomness inherent in Monte Carlo sampling, a single run of CARS exhibits considerable stochastic variability, leading to inconsistent wavelength selection results across repeated experiments. This undermines the stability and reproducibility of detection models. In recent years, swarm intelligence optimization algorithms—such as particle swarm optimization, genetic algorithms, and the sparrow search algorithm (SSA)—have been widely adopted in the field of feature selection due to their strong global search capabilities and ability to avoid local optima [17].

Partial least squares regression (PLSR), a linear model, has remained a dominant approach in NIR spectral analysis due to its strong stability and excellent interpretability. The applicability of linear models for NIR spectral detection has been well established across various cereal crops. Zhang et al. applied PLSR to detect starch content in corn, achieving a test R^2^ of 0.78 and validating the reliability of linear models for routine quality detection tasks in this crop [18]. An et al. noted that PLSR effectively reduces collinearity in high-dimensional spectral data while preserving model interpretability, which has led to its widespread use in cereal quality analysis [19]. Studies have applied PLSR to detect protein content in wheat, achieving a test R^2^ of 0.92 and further confirming the model’s generalizability [20]. Additionally, Wang et al. emphasized that the simple structure and ease of implementation of linear models have underpinned their longstanding dominance in NIR spectral analysis, as demonstrated by their successful application in quality detection for oats and sorghum [21]. However, studies have shown that the relationship between spectral features and component content often contains nonlinear components that linear models struggle to fully capture. Xu et al. found in their research on corn starch detection that the absorption of near-infrared light by starch is influenced by interactions among multiple components (such as moisture and protein), resulting in a nonlinear relationship between spectral signals and starch content [22]. Under such circumstances, PLSR models tend to either underestimate or overestimate the actual content. Sampaio et al. pointed out that linear models often have limited fitting accuracy when dealing with complex spectral systems, such as those involving amylose and amylopectin content in rice, and are unable to fully exploit the latent information embedded in spectral data [23]. They further noted that this limitation is also evident in wheat and barley, where linear models struggle to capture the nonlinear relationships between spectral features and key quality indicators.

Nonlinear models possess stronger fitting capabilities and can better capture nonlinear relationships among variables, a fact that has been validated across multiple cereal crops. Liu et al. developed an artificial neural network (ANN) model for predicting corn starch content [24]. Compared with the PLSR model, the ANN achieved a 0.12 increase in test R^2^, demonstrating the advantage of nonlinear models in fitting complex relationships. Almoujahed et al. applied gradient boosting decision tree (GBDT) to the NIR spectroscopic analysis of wheat gluten content and found that it effectively reduced the impact of anomalous data while improving model robustness [25]. This approach was extended to barley with similarly positive results. However, the generalization performance of these nonlinear models under small-sample conditions still requires further validation across different cereal crops. Mansuri et al. pointed out that when the sample size is fewer than 100, ANN is prone to overfitting in both corn and rice detection studies [26]. In wheat and oat detection, GBDT may exhibit poor generalization under small-sample conditions due to excessive reliance on training data features. Convolutional neural networks (CNNs), which have achieved remarkable success in image recognition, have gradually been applied to NIR spectral analysis of various cereals in recent years. For detecting amylose content in rice, Li et al. noted that CNNs, originally designed for two-dimensional image data, often require large numbers of samples when applied to one-dimensional spectral data [27]. This limitation has also been observed in NIR spectral studies of wheat and barley. Furthermore, most current research focuses on improving test accuracy, while relatively little attention has been given to the chemometric interpretation of the selected characteristic wavelengths. Integrating data-driven feature selection results with molecular vibrational spectroscopy theory, assigning clear chemical bond assignments and physical meanings to characteristic wavelengths, not only enhances model interpretability but also helps validate the rationality and effectiveness of the feature selection outcomes.

From 59 initially collected corn varieties, one was excluded via quality control and outlier detection, yielding 58 varieties for model development and evaluation. Their starch contents were determined using the DNS colorimetric method, and NIR spectral data were acquired over the wavelength range of 950–1650 nm. A three-step combined preprocessing strategy was applied to the spectral data. A two-layer feature screening framework, consisting of CARS for coarse selection and SSA for fine selection, was then constructed to extract key wavelengths. Based on these selected wavelengths, the predictive performances of four models were systematically compared: PLSR, ANN, CNNs, and GBDT. The objectives of this study are to: (1) identify characteristic wavelengths that are highly correlated with corn starch content and provide mechanistic interpretations of their chemical bond assignments; (2) compare the adaptability of different model types to spectral data and reveal their performance differences in spectral analysis tasks; and (3) establish a high-precision, high-stability rapid detection model for corn starch content, thereby providing technical support for corn quality evaluation, breeding selection, and grading of processing raw materials.

## 2. Materials and Methods

### 2.1. Experimental Design and Material Collection

A total of 59 corn varieties, including both locally cultivated and introduced demonstration varieties, were used as test materials. The variety details are provided in Table 1. The experiment was conducted in Sijiachi Village, Longquan Town, Huguan County, Changzhi City, Shanxi Province (36.08° N, 113.02° E). This area is a typical rainfed agricultural region, characterized by cinnamon soil that is deep, well-developed, and moderately fertile. The experimental field had flat terrain and uniform soil moisture conditions. Mechanized sowing was completed using a dryland corn soil moisture-detecting seeder on 8 May 2025. Prior to sowing, straw crushing and returning to the field, as well as fine soil preparation, were carried out with a plowing depth of 15–20 cm to ensure a flat surface and fine soil clods. During sowing, the soil temperature at the 5 cm depth was stabilized above 10 °C, the sowing depth was controlled at 3–4 cm, and the planting density was 3500–4000 plants per mu, which complied with the local dryland corn planting specifications. A compound fertilizer containing controlled-release nitrogen (N-P_2_O_5_-K_2_O ratio of 26-13-6) was applied as base fertilizer at a rate of 45 kg per mu. During sowing, the fertilizer was side-deep placed at a depth of 10 cm, 7–10 cm outside the seeding furrow. During the growing season, two rounds of unmanned aerial vehicle applications were carried out for pest and disease control, depending on soil moisture conditions. Foliar fertilizer and low-toxic pesticides were applied on a regular basis to control pests and diseases such as corn borers and northern leaf blight in corn. These measures also helped alleviate soil compaction, conserve soil moisture, and improve water retention.

Harvesting of all variety plots was completed on 15 October 2025, at which time the corn kernels had reached physiological maturity, as indicated by the disappearance of the milk line, the formation of the black layer, and the loosening of the pericarp. Sampling was conducted in accordance with the random sampling principle, with 3 replicates set for each variety. Plump kernels free from diseases and insect pests were selected, and each sample was accurately weighed to 100 g. The samples were naturally air-dried in a well-ventilated and sunny location. During the air-drying period, the samples were turned regularly, and the environmental conditions were controlled at a temperature of 25–30 °C and a relative humidity of 50–60%. Air-drying was continued until the kernel moisture content dropped below 13%, after which the samples were hermetically sealed and stored in a dry, well-ventilated sample cabinet for subsequent use.

### 2.2. Near-Infrared Hyperspectral Imaging Acquisition

Corn samples were placed into containers with a diameter of 5 cm and a depth of 3 cm. After uniform filling, compaction, and surface leveling, near-infrared hyperspectral images were acquired using a push-broom hyperspectral imager (Headwall Photonics, Hexagon AB, North Kingstown, RI, USA). The instrument has a spectral range of 900–1700 nm, comprising 172 bands with a spectral resolution of 4.715 nm. During data acquisition, the distance between the lens and the sample was fixed at 280 mm, and the scanning speed was set to 2.721 mm/s. To reduce noise caused by dark current at the two ends of the spectrum (<950 nm and >1650 nm), and to improve the stability of the model data and the reliability of the selected features, this study limited the effective modeling range to 950–1650 nm, corresponding to 148 wavelengths [17]. This spectral range is suitable for establishing a quantitative hyperspectral detection model for corn starch content. After preparation, each sample was measured three times consecutively, and the average spectrum was used for subsequent analysis.

### 2.3. Starch Content Determination

The starch content of corn samples was determined using the enzymatic hydrolysis method specified in the Chinese national standard GB 5009.9-2016 [28]. The principle of this method involves the removal of fat and soluble sugars from the samples, followed by sequential two-step enzymatic hydrolysis to completely convert starch into glucose [7,8,9,10]. The reducing sugar content was then determined by titration, and the starch content was calculated by multiplying the glucose equivalent by a conversion factor of 0.90.

Corn kernels were ground to pass through a 40-mesh sieve, thoroughly mixed, and stored in sealed containers until analysis. An aliquot of 2.000 g of the ground sample was weighed and defatted by successive washing with petroleum ether. Soluble sugars were then removed by washing with 85% ethanol solution to eliminate interference from free sugars in the subsequent detection. After the solvent was fully drained, the pretreated sample residue was transferred to a beaker, and an appropriate volume of warm water was added. The mixture was heated in a boiling water bath for 15 min to fully gelatinize the starch. After cooling to below 60 °C, α-amylase solution was added, and the mixture was incubated at 55–60 °C for 60 min to liquefy the starch. Complete liquefaction was confirmed by the absence of blue coloration upon addition of iodine solution. The enzyme was then inactivated by boiling, and the sample solution was cooled to room temperature. The pH of the solution was adjusted to 4.5–5.0, followed by the addition of glucoamylase solution. The mixture was incubated at 60 °C for 2 h to ensure complete hydrolysis of intermediate dextrins and other oligosaccharides into glucose. After cooling, the sample solution was transferred to a 250 mL volumetric flask, made up to volume with distilled water, and thoroughly mixed. The solution was filtered through dry filter paper, and the initial filtrate was discarded. A blank test was conducted in parallel to correct for systematic errors. The glucose content in the sample filtrate was determined using the alkaline copper tartrate direct titration method, which involved separate standardization of the titrant and titration of the sample filtrate. Finally, the mass fraction of starch in the corn sample was calculated according to the formula provided in the national standard, taking into account the sample weight, dilution volume, and titration results.

### 2.4. Spectral Data Preprocessing

Wavelet transform (WT) [29] decomposes spectral signals into frequency bands at different scales, enabling the separation of noise from useful signals. Its core lies in threshold processing. In this study, the Penalty strategy was adopted to adaptively determine the threshold size, effectively filtering out high-frequency noise while preserving useful spectral features. This strategy balances signal fidelity and smoothness, automatically selecting the decomposition level and threshold through a penalty function, thereby avoiding human intervention. Specifically, the parameters are: mother wavelet bior3.3; decomposition level lev = 5; penalty strategy for threshold selection with alpha = 2; noise standard deviation estimated via wnoisest from level-1 detail coefficients; soft thresholding (‘s’); global thresholding mode (‘gbl’); and approximation coefficients retained (keepapp = 1).

Multiplicative Scatter Correction (MSC) [30] assumes a linear relationship between each sample spectrum and an ideal spectrum (typically taken as the mean spectrum of all samples). By performing linear regression to fit each sample spectrum against the mean spectrum, MSC corrects the offset and slope, thereby eliminating spectral baseline drift and intensity variations caused by physical factors such as particle size and surface scattering. This enhances the absorption features related to chemical composition.

Standard Normal Variate (SNV) [31] transformation processes each sample spectrum individually by calculating the mean and standard deviation across all wavelengths of that spectrum and then standardizing it. Its purpose is to eliminate multiplicative scattering effects caused by sample heterogeneity and path length variations, while also removing baseline shifts. This makes the spectra of different samples comparable, making SNV particularly suitable for correcting reflectance spectra of solid samples.

To ensure the effectiveness of model training, the objectivity of validation, and the generalizability of test, this study employed the SPXY algorithm [32], based on the joint distribution of spectral data and quality indicators, to partition the samples into training, validation, and test sets with a ratio of 6:2:2. Specifically, 60% of the samples were used as the training set for feature learning and parameter training. Among the remaining samples, 20% were selected as the validation set for hyperparameter optimization and iterative screening. The final 20% served as the test set for unbiased evaluation of the final model performance. By applying the principle of joint distance maximization for sample selection, the three datasets maintain consistent distribution characteristics in both spectral features and target attributes, thereby avoiding model overfitting or insufficient generalization due to sample distribution bias.

### 2.5. Key Wavelength Extraction

To address the issues of redundant variables and strong collinearity inherent in high-dimensional near-infrared spectra, a key wavelength extraction strategy was developed, combining preliminary screening using Competitive Adaptive Reweighted Sampling (CARS) [15,33] with fine screening using the Sparrow Search Algorithm (SSA) [17,34]. This approach reduces data dimensionality while improving model test accuracy and computational efficiency.

First, the CARS algorithm was used for preliminary screening of high-frequency wavelengths. This algorithm combines Monte Carlo sampling with partial least squares (PLS) regression coefficients. It selects characteristic wavelengths with large absolute regression coefficients through an exponentially decreasing decay function, eliminating uninformative and redundant variables. In this study, the maximum number of principal components for PLS was set to 10, and the optimal number of principal components was determined through 10-fold cross-validation on the training set. To evaluate wavelength stability, the CARS algorithm was run 500 times independently, each with a different random seed. Within each CARS run, the number of Monte Carlo sampling iterations was set to 1000 to ensure adequate sampling coverage and feature selection stability. Model error was calculated using 10-fold cross-validation and used as the evaluation criterion for feature selection. The selection frequency of each wavelength variable was recorded across all 500 independent runs. Wavelengths with a selection frequency greater than 30% were included in the candidate wavelength set. This threshold was determined empirically based on the natural breakpoint observed in the selection frequency distribution, ensuring that only wavelengths with consistently high stability were retained while excluding those selected sporadically due to random sampling variability.

Next, the Sparrow Search Algorithm (SSA) was used to optimize the selection of candidate features. SSA simulates the foraging and anti-predation behavior of sparrow populations, offering strong global search capability, fast convergence, and high optimization precision. It effectively overcomes the limitation of traditional algorithms that tend to get trapped in local optima. Binary encoding was used to implement 0–1 feature selection, with parameters balanced between optimization accuracy and computational efficiency. Specifically, the sparrow population size was set to 20, and the maximum number of iterations was set to 50. During the discoverer update process, a random number was used as the warning threshold. When the random number was less than 0.8, an adaptive position update was performed; otherwise, a random perturbation update was applied. The continuous optimization solutions were converted into binary feature selection results using a sigmoid function. The fitness function was defined as the minimum root mean square error of cross-validation (RMSECV) from partial least squares with 10-fold cross-validation, aiming to minimize the fitness value to select the optimal set of key wavelengths.

### 2.6. Model Construction

The core of PLSR [16,18,19,20] lies in simultaneously performing dimensionality reduction and regression. By iteratively extracting latent variables from the independent variable matrix and the dependent variable, PLSR maximizes the covariance between the latent variables and the dependent variable, thereby filtering out noise while retaining key information. Ultimately, a regression model is established using a small number of latent variables, effectively addressing the issues of the curse of dimensionality and collinearity. Data centering was used as the preprocessing method, and the number of principal components was searched within a range of 1 to 15. The optimal number of principal components was determined using 10-fold cross-validation. The RMSECV was calculated for models with different numbers of principal components, and the number corresponding to the minimum RMSECV was selected as the optimal value, balancing model fitting ability and generalization performance.

The core of ANN [24,26,35] lies in the nonlinear fitting and end-to-end mapping of a multi-layer fully connected neural network. The feature vector composed of the selected characteristic spectral bands is input into the network. Through the weight matrices of each layer, the effective bands of the characteristic spectrum are integrated in a weighted manner. Combined with a nonlinear activation function, the complex nonlinear quantitative relationship between the characteristic spectrum and starch content is uncovered. A single hidden-layer feedforward structure was adopted, and the number of hidden-layer nodes was optimized. The training function employed the Bayesian regularization algorithm, with a maximum number of iterations of 1000 and a minimum training gradient of 1 × 10^−7^.

The core of CNNs [27,36] lies in adapting to the one-dimensional sequential characteristics of the feature spectrum. By sliding one-dimensional convolution kernels along the wavelength dimension of the feature spectrum, the CNNs accurately extract local correlation features among adjacent characteristic bands and the combination patterns of characteristic absorption peaks. Weight sharing reduces the number of model parameters, while pooling layers further reduce dimensionality and remove noise from the convolutional features, filtering out measurement redundancy and noise interference from the feature spectrum. Through the stacking of multiple convolutional layers, the core spectral features strongly correlated with starch content are progressively distilled. Finally, the predicted starch content is output through a fully connected layer. A single-channel, one-dimensional input structure was adopted. The convolutional kernel size was set to 5 × 1, and the number of convolutional kernels was optimized. The number of nodes in the fully connected layer was also optimized. The activation function used was ReLU, with a pooling kernel size of 2 × 1 and a stride of 2. The optimizer was Adam, with a maximum number of iterations of 1000 and an L2 regularization coefficient of 0.001. An early stopping patience of 8 was set to prevent overfitting.

The core of GBDT [25,37] lies in its forward stage-wise iterative approach to residual fitting using an additive model. Using the CART regression tree as the base learner, each effective wavelength of the feature spectrum is treated as an input feature. Starting from an initial simple regression tree, each subsequent iteration trains a new decision tree to fit the residual error of the starch content test from the previous model. By continuously compensating for test errors, the model gradually improves its accuracy. Finally, the weighted sum of the outputs from all weak regression trees yields the final predicted value. The LSBoost least squares boosting strategy was adopted, with decision trees as the base learners. The maximum number of splits was set to 12, and both the number of decision trees and the learning rate were optimized. A grid search was used to traverse all parameter combinations, and the validation set RMSE was calculated for each combination to determine the optimal number of trees and learning rate. Model stability was further evaluated using 10-fold cross-validation.

To objectively evaluate model performance and stability, all prediction models were independently run 50 times to eliminate the influence of random errors. PLSR is a deterministic modeling method, where training and testing are performed under fixed optimal principal components. In contrast, CNNs, ANN, and GBDT reinitialize either the network weights or the base learner structure in each run, thereby fully capturing the random fluctuations and generalization differences in the models under different initial conditions. The evaluation metrics for the training, validation, and test sets included the coefficient of determination (R^2^), RMSE, and relative percent deviation (RPD) [17,23,35,36,37]. R^2^ indicates the model’s goodness of fit, RMSE represents the deviation between predicted and true values, and RPD reflects the model’s application potential.

## 3. Results

### 3.1. Analysis of Starch Content in Different Corn Varieties

**Figure 1:**
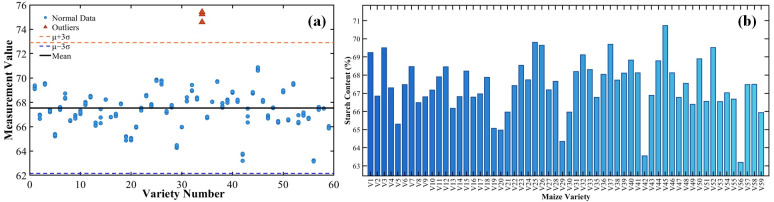
Outlier detection was performed on the starch content of 59 corn varieties, followed by differential analysis after the outliers were removed. (**a**) Outlier analysis using the 3σ criterion; (**b**) Difference analysis of starch content after removing outlier varieties.

Figure 1a illustrates the detection of outliers in the original starch content data of 177 corn samples using the 3σ criterion [38,39]. Statistical results show an overall mean of 67.527% and a standard deviation of 1.791%. Based on these values, the upper and lower 3σ thresholds were calculated as 72.900% and 62.156%, respectively. Three outliers were identified, all from variety V34 and all exceeding the upper threshold. Given the unique genetic characteristics of the V34 parental line—which may contribute to its unusually high starch accumulation—this variety was excluded to ensure the robustness of subsequent statistical analyses. The remaining dataset comprised 58 normal corn varieties (V1–V33, V35–V59), and the average of three replicate measurements per variety was used as the representative starch content. As shown in Figure 1b, starch content varied significantly among varieties. Variety V45 had the highest mean value (70.720%), while variety V56 had the lowest (63.193%). The remaining varieties ranged from 64.350% to 69.807%, indicating substantial genetic diversity.

Further statistical analysis was conducted on the 58 retained varieties (174 samples in total). The overall mean starch content was 67.397%, with a standard deviation of 1.504% and a median of 67.477%. The coefficient of variation was 2.231%, well below 10%, indicating low data dispersion and good repeatability. The skewness coefficient was −0.474, showing a slightly left-skewed distribution. The kurtosis coefficient was 3.520, indicating a leptokurtic distribution. The 95% confidence interval ranged from 67.002% to 67.793%, a relatively narrow interval suggesting high precision in the mean estimate. The Kolmogorov–Smirnov test (h = 0, *p* = 0.782 > 0.05) supported that, at the 95% confidence level, the starch content data of the corn varieties followed a normal distribution.

### 3.2. Analysis of Corn Spectral Response and Preprocessing

**Figure 2:**
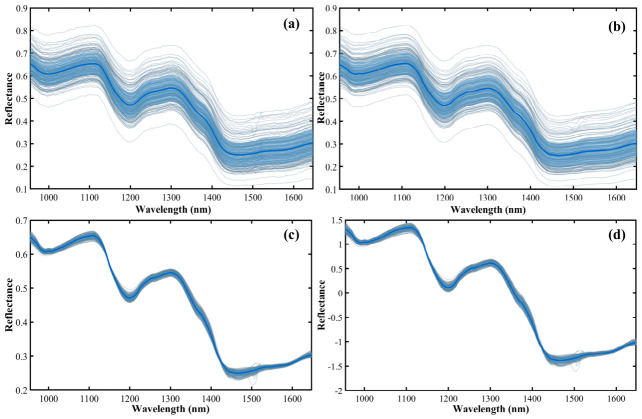
Near-infrared spectral responses and preprocessing results for 174 corn kernels. (**a**) Original spectra; (**b**) WT preprocessing; (**c**) WT + MSC preprocessing; (**d**) WT + MSC + SNV preprocessing. (Note: Dark gray lines represent the spectral responses of individual samples; the dark blue line represents the overall average spectrum of all samples; the shaded areas above and below the dark blue line indicate the spectral standard deviation).

Figure 2a shows the near-infrared reflectance spectra of the corn samples, where reflectance exhibits certain fluctuating patterns with changing wavelength. In the ranges of 950–1000 nm, 1100–1200 nm, and 1300–1450 nm, reflectance shows a decreasing trend, indicating relatively strong absorption of near-infrared light by corn in these regions. This may be associated with overtone or combination absorptions resulting from the stretching and bending vibrations of chemical bonds in components such as starch, protein, water, and fat. In contrast, within the ranges of 1000–1100 nm, 1200–1300 nm, and 1450–1650 nm, reflectance generally shows an increasing trend, suggesting relatively weak light absorption in these regions. This may be attributed to the surface scattering effects of corn kernels or to the overlap and cancellation of absorption bands from different chemical components within these spectral intervals [40].

After WT processing (Figure 2b), the spectral curves maintained a similar overall trend to the original spectra, but with clearer detailed features, indicating that wavelet transform effectively filtered out high-frequency noise. Following the addition of MSC (Figure 2c), the parallelism between the curves improved, reflecting that MSC corrected the light scattering effects caused by variations in particle size and uneven distribution, thereby enhancing the correlation between the spectra and chemical composition. Finally, after the combined WT + MSC + SNV preprocessing (Figure 2d), the spectral data were then subjected to SNV transformation. This correction effectively eliminates baseline shifts and optical path differences among samples, making the spectral data of different samples more comparable. MSC corrects based on inter-sample relationships, while SNV corrects based on intra-sample characteristics; their combination provides a more comprehensive removal of scattering effects. The results for the training, validation, and test sets are presented in Table 2.

### 3.3. Key Wavelength Selection Results

**Figure 3:**
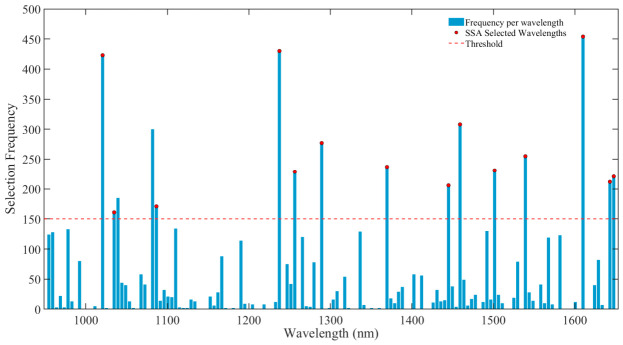
Sensitive wavelengths related to starch content in corn kernels selected by the CARS and SSA algorithms.

CARS is a feature selection method based on the ensemble modeling concept. Its core criterion is the RMSECV. By constructing a multi-model ensemble system, CARS iteratively eliminates invalid wavelengths that are weakly correlated with corn starch content or have low contribution, retaining the core features that strongly explain the target variable. As irrelevant wavelengths are gradually removed, redundant information in the model decreases. Consequently, the fitting accuracy for starch content continuously improves, and the corresponding RMSECV value shows a declining trend. When the RMSECV value reaches its minimum, the retained set of wavelengths is considered the initial optimal feature band most highly correlated with corn starch. CARS was run for 500 iterations, and a threshold of 30% was applied to constrain and validate the feature selection results. Ultimately, a candidate set of 16 characteristic wavelengths was preliminarily screened, as shown in Figure 3. To further verify whether any residual redundancy remained in the CARS-selected results, SSA was introduced to validate and optimize the 16 candidate wavelengths. The results show that after optimization by the SSA algorithm, 14 characteristic wavelengths highly correlated with corn starch content were finally selected from the 16 candidates. This indicates that redundancy still existed in the wavelength set from the coarse CARS screening. The optimized wavelengths are 1020.65, 1034.80, 1086.66, 1237.53, 1256.39, 1289.39, 1369.54, 1444.97, 1459.12, 1501.55, 1539.27, 1609.99, 1642.99, and 1647.71 nm. The corresponding optimization results are shown in Figure 3. These results fully demonstrate that a small amount of redundant information exists in the original spectral features, and not all wavelengths contribute effectively to the detection of corn starch content.

### 3.4. Results of Model Construction

Four predictive models, PLSR, ANN, CNNs, and GBDT, were constructed in this study. Variables were extracted from the validation set based on the selected set of key wavelengths, and model parameters were optimized using the validation set data. The optimal number of principal components for PLSR was 7, with a corresponding RMSECV of 1.227%. For the CNNs, after 100 iterations with 20 sub-iterations each, the optimal structure consisted of 16 convolutional kernels and 32 fully connected nodes, achieving an RMSECV of 1.227%. The optimal number of hidden layer nodes for the ANN was 10. For the GBDT model, through a grid search over combinations of tree numbers and learning rates, the optimal parameter combination was 200 trees and a learning rate of 0.05, yielding an RMSECV of 0.791%. To further evaluate the generalization ability of the optimal GBDT model, 10-fold cross-validation was conducted. Each model was run 50 times, and the results are presented in Table 3.

Based on the evaluation metrics for the training, validation, and test sets, significant differences in fitting ability and generalization performance were observed among the models. The overall ranking was ANN > GBDT > PLSR > CNNs. On the training set, the GBDT model achieved the best performance, with an R^2^ of 0.877, which is approximately 22.6% higher than that of PLSR and 71.3% higher than that of CNNs. Its RMSE was only 0.724%, representing reductions of 8.2% and 31.7% compared with PLSR and CNNs, respectively, and it attained an RPD of 2.85. These results indicate that ensemble learning can efficiently capture the nonlinear relationships between spectral data and target values. The ANN model performed close to GBDT, with a training set R^2^ of 0.867, approximately 21.3% higher than PLSR, demonstrating excellent nonlinear fitting capability. In contrast, the CNNs achieved a training set R^2^ of only 0.512, significantly lower than the other models, indicating that one-dimensional spectral data are less compatible with basic CNN architectures.

On the test set, the GBDT model achieved an R^2^ of 0.693, which is approximately 7.0% higher than that of PLSR and 57.1% higher than that of CNNs. Its RMSE was 0.809%, and the RPD was 1.80. The ANN model achieved a test R^2^ of 0.826, approximately 18.0% higher than GBDT, with an RMSE reduced to 0.759% and an RPD of 2.40, making it the best-performing model in terms of predictive capability and highlighting the advantages of deep feature learning. The PLSR model performed steadily, with a test R^2^ of 0.648 and an RPD of 1.69, aligning with the baseline level expected of linear models. The CNN achieved a test R^2^ of only 0.441 and an RMSE of 1.521%, far from meeting practical requirements.

## 4. Discussion

The 58 corn varieties showed significant starch content variation, ranging from 63.193% (V56) to 70.720% (V45)—a difference of 7.527 percentage points. This genetic diversity provided an ideal data foundation for building generalizable predictive models. After removing three outliers from variety V34 using the 3σ criterion, the starch content data exhibited good normality (*p* = 0.782). The SPXY sample partitioning method ensured consistent distributions across training, validation, and test sets. Their mean starch contents were 67.415%, 67.332%, and 67.409%, with standard deviations of 1.529%, 1.726%, and 1.183%, respectively. The slightly higher standard deviations in the validation and test sets indicated broader content coverage, which helped comprehensively assess model generalization. Overall, the high-quality data partitioning supported stable model training and reliable evaluation.

The variation in starch content among different corn varieties reflects differences in the genetic regulation of starch synthesis and accumulation in corn kernels. Starch consists of amylose and amylopectin, and the observed differences in starch content are primarily determined by the activities of key enzymes such as ADP-glucose pyrophosphorylase, starch synthase, and starch branching enzyme [41,42]. Differences in the expression levels of these enzymes not only directly affect the final starch content but may also indirectly influence near-infrared spectral response characteristics by altering kernel structure, moisture distribution, and the protein matrix. Among the 14 characteristic wavelengths identified in this study, wavelengths at 1444.97 nm and 1459.12 nm fall within the region associated with the first overtone of the O–H bond, which is a major constituent of hydroxyl groups in starch molecules [43,44]. However, it is important to note that NIR absorption bands are inherently broad and overlapping, and contributions from water (also containing O–H bonds) and other matrix components cannot be excluded. Consequently, these wavelengths are best interpreted as likely reflecting starch-related chemical information, rather than as uniquely diagnostic of starch. Meanwhile, wavelengths at 1501.55 nm and 1539.27 nm lie within the N–H bond absorption region and may indirectly reflect starch variation through their correlation with protein content [45,46], as starch and protein synthesis are metabolically interconnected during kernel development. The remaining characteristic wavelengths, while not directly attributable to specific starch-related bonds, may capture variations in physical properties such as kernel density, moisture distribution, and scattering effects that are influenced by starch content. The availability of both direct absorption-related signals and indirect correlated signals provides rich information redundancy for the model, which is a key reason why nonlinear models such as ANN and GBDT achieve excellent predictive performance.

The comparison among models revealed notable differences in predictive performance, which may provide insights into the nature of the relationship between spectral features and starch content. The observation that ANN (R^2^ = 0.826) and GBDT outperformed PLSR (R^2^ = 0.648) suggests that the relationship may involve complex patterns that are not entirely captured by linear models. This inference is consistent with the inherent capacity of tree-based and neural network models to capture feature interactions and nonlinear dependencies through hierarchical transformations. However, it is important to note that the performance advantage of ANN and GBDT over PLSR does not directly quantify the degree of nonlinearity present in the data, as other factors—including differences in model architecture, parameter optimization, noise tolerance, and the ability to handle multicollinearity among spectral predictors—may also contribute to the observed performance gap. Meanwhile, the CNN model exhibited substantially lower predictive performance (R^2^ = 0.441) compared with both ANN and GBDT. This discrepancy may be attributable to the relatively limited sample size (174 samples), which is insufficient to fully train deep learning architectures that typically require large-scale datasets to learn hierarchical feature representations effectively. Given the modest dataset, the CNN model likely suffered from overfitting or inadequate feature learning, despite the regularization strategies implemented. Collectively, these results indicate that while the spectral–composition relationship in this dataset is not sufficiently complex to justify the use of sophisticated deep learning architectures, the moderate improvement of ANN and GBDT over PLSR suggests that nonlinear modeling approaches can provide added value by exploiting subtle patterns that linear models may overlook. The remaining variance not accounted for by PLSR may stem from factors such as nonlinear scattering effects caused by differences in kernel structure among varieties, as well as coupling effects from protein–starch interactions. These findings highlight the importance of selecting appropriate model complexity in relation to dataset size and the intrinsic complexity of the underlying biological system.

The 14 characteristic wavelengths identified in this study cover multiple spectral bands directly related to starch (O–H and C–H) as well as indirectly related bands (N–H). This result provides a reference for wavelength selection in the development of portable near-infrared detection devices. In practical applications, dedicated detection modules can be designed based on these key wavelengths, significantly reducing equipment costs and enabling rapid, non-destructive screening of starch content in corn varieties. Furthermore, the excellent performance of the GBDT and ANN models on the test set (RPD of 1.80 and 2.40, respectively) indicates that prediction models based on these key wavelengths have reached a preliminary level of practical utility and can provide technical support for the early selection of high-starch varieties in corn breeding.

Although this study covered 58 varieties, the samples were primarily collected from a single ecological region and growing season, which may not fully capture the influence of environmental factors (such as light, temperature, and soil conditions) on starch content. Corn starch content is significantly affected by genotype-by-environment interactions; the same variety may exhibit differences in starch content across planting years or geographic regions. Future research should expand the temporal and spatial dimensions of the sample set by incorporating multi-year, multi-location trial data to evaluate the environmental robustness of the models. Furthermore, integrating genomic information to develop a multi-modal “genotype–spectrum–phenotype” prediction model holds promise for further improving prediction accuracy and deepening our understanding of the mechanisms underlying starch accumulation.

## 5. Conclusions

In this study, 58 corn varieties were used to systematically investigate a rapid detection method for starch content based on near-infrared spectroscopy. A three-stage sequential preprocessing strategy—wavelet transform, multiplicative scatter correction, and standard normal variate transformation—was applied. This effectively filtered out high-frequency noise, corrected particle scattering effects, eliminated baseline drift, and significantly enhanced the correlation between spectra and starch content. A two-layer screening strategy consisting of CARS coarse selection followed by SSA fine selection was established, identifying 14 key characteristic wavelengths. These wavelengths cover O–H and C–H absorption regions directly related to starch, as well as the N–H region indirectly associated with starch, offering good chemometric interpretability. Among the prediction models, ANN performed best, achieving an R^2^ of 0.826, RMSE of 0.759%, and RPD of 2.40 on the test set, demonstrating strong nonlinear mapping capability. GBDT ranked second (R^2^ = 0.693, RPD = 1.80), validating the effectiveness of ensemble learning in spectral analysis. PLSR, as a linear benchmark model, performed steadily (R^2^ = 0.648, RPD = 1.69). In contrast, CNNs exhibited poor predictive performance (R^2^ = 0.441) due to a mismatch between model complexity and data characteristics under small-sample conditions. This study provides a methodological reference for rapid detection of corn starch content, and the identified key wavelengths can support the development of portable detection devices. The findings have practical value for corn quality breeding and the screening of processing raw materials.

## Figures and Tables

**Table 1:** Information on 59 rainfed corn varieties.

Varieties	Variety Names	Varieties	Variety Names	Varieties	Variety Names	Varieties	Variety Names
V1	T3307K	V16	Chengdan 1	V31	Keyu 27	V46	Huiyu 618
V2	SLH333	V17	BK9616	V32	Yida 126	V47	G9922K
V3	Liaodan 318	V18	Fenghe 2002	V33	Hongbo 692	V48	Jinyuan 59
V4	A6017	V19	Menglong 213	V34	TF9618	V49	TLY202
V5	Shennong 4200	V20	Fadi 868	V35	HB9312	V50	Fengke 950
V6	Wanda 56	V21	Qiansong 1507	V36	Keyu 2020	V51	Chongli 218
V7	Qiankun 2202	V22	Lanqiao 8848	V37	DN4018	V52	Quanyan 518
V8	Runmin 2308	V23	Luyu 711	V38	Dongdan 3000	V53	Zhong’ou 6
V9	Huawei 1	V24	Zhenghong T55	V39	LG4363	V54	Zhongbang 202
V10	Ouya 91	V25	Bangni 178	V40	Tie 3135	V55	Zhongbang 3K1
V11	WK1505	V26	Wufu AK47	V41	Yuanlihe 619	V56	Zhongbang 398
V12	Liaoshenghe 198	V27	Wufu 818	V42	Fuyuan Yu 999	V57	Zhongmiao 6S
V13	KF3699	V28	Wanhong 138	V43	Pengyu 18	V58	Haicang 528
V14	Ruifeng 606	V29	Xianyu 335	V44	Taifeng 55	V59	Fengtian 801
V15	Dongke 1188	V30	Wanhong 137	V45	Qingdan 18		

**Table 2:** Results of statistical dataset partitioning (%).

Sample Set	Number of Samples	Maximum	Minimum	Mean	Standard Deviation
Training	104	70.870	63.140	67.415	1.529
Validation	35	70.690	63.220	67.332	1.726
Test	35	69.860	65.060	67.409	1.183

**Table 3:** Results of the four prediction models for starch content in corn kernels.

Models	Training Set			Validation Set			Test Set		
	R^2^	RMSE (%)	RPD	R^2^	RMSE (%)	RPD	R^2^	RMSE (%)	RPD
PLSR	0.715	0.788	1.87	0.709	0.799	1.855	0.648	0.831	1.69
ANN	0.867 ± 0.0466	0.729 ± 0.0641	2.74 ± 0.204	0.852 ± 0.0481	0.739 ± 0.0683	2.60 ± 0.162	0.826 ± 0.0588	0.759 ± 0.0706	2.40 ± 0.0127
CNNs	0.512 ± 0.0121	1.06 ± 0.0114	1.43 ± 0.031	0.479 ± 0.0114	1.23 ± 0.0092	1.39 ± 0.0253	0.441 ± 0.0237	1.52 ± 0.0136	1.34 ± 0.0024
GBDT	0.877	0.724	2.85	0.722	0.791	1.90	0.693	0.809	1.80

## Data Availability

The original contributions presented in the study are included in the article; further inquiries can be directed to the corresponding author.

## Abbreviations

The following abbreviations are used in this manuscript:

NIR	Near-infrared
CARS	Competitive adaptive reweighted sampling
PLS	Partial least squares
SSA	Sparrow search algorithm
PLSR	Partial least squares regression
ANN	Artificial neural network
GBDT	Gradient boosting decision tree
CNNs	Convolutional neural networks
WT	Wavelet transform
MSC	Multiplicative scatter correction
SNV	Standard normal variate
CV	Coefficient of Variation
R^2^	Coefficient of determination
RMSE	Root mean square error
RPD	Relative Percent Deviation
